# Structure of receptive fields in a computational model of area 3b of primary sensory cortex

**DOI:** 10.3389/fncom.2014.00076

**Published:** 2014-07-28

**Authors:** Georgios Is. Detorakis, Nicolas P. Rougier

**Affiliations:** ^1^Laboratoire des Signaux et Systèmes, SupélecGif-sur-Yvette, France; ^2^INRIA Bordeaux Sud-OuestBordeaux, France; ^3^Institut des Maladies Neurodégénératives, Université de Bordeaux, Centre National de la Recherche Scientifique, UMR 5293Bordeaux, France; ^4^LaBRI, Université de Bordeaux, Institut Polytechnique de Bordeaux, Centre National de la Recherche Scientifique, UMR 5800Talence, France

**Keywords:** receptive field, neural field, somatosensory cortex, area 3b, SI, computational model, self-organization

## Abstract

In a previous work, we introduced a computational model of area 3b which is built upon the neural field theory and receives input from a simplified model of the index distal finger pad populated by a random set of touch receptors (Merkell cells). This model has been shown to be able to self-organize following the random stimulation of the finger pad model and to cope, to some extent, with cortical or skin lesions. The main hypothesis of the model is that learning of skin representations occurs at the thalamo-cortical level while cortico-cortical connections serve a stereotyped competition mechanism that shapes the receptive fields. To further assess this hypothesis and the validity of the model, we reproduced in this article the exact experimental protocol of DiCarlo et al. that has been used to examine the structure of receptive fields in area 3b of the primary somatosensory cortex. Using the same analysis toolset, the model yields consistent results, having most of the receptive fields to contain a single region of excitation and one to several regions of inhibition. We further proceeded our study using a dynamic competition that deeply influences the formation of the receptive fields. We hypothesized this dynamic competition to correspond to some form of somatosensory attention that may help to precisely shape the receptive fields. To test this hypothesis, we designed a protocol where an arbitrary region of interest is delineated on the index distal finger pad and we either (1) instructed explicitly the model to attend to this region (simulating an attentional signal) (2) preferentially trained the model on this region or (3) combined the two aforementioned protocols simultaneously. Results tend to confirm that dynamic competition leads to shrunken receptive fields and its joint interaction with intensive training promotes a massive receptive fields migration and shrinkage.

## Introduction

In a previous work (Detorakis and Rougier, 2012), we proposed a computational model of the somatosensory cortex based on neural field theory (Amari, 1977; Bressloff, 2011). This model allowed us to investigate formation and maintenance of ordered topographic maps in the primary somatosensory cortex during the critical period of development (postnatal), where representations are shaped, and the post-critical period, where representations are maintained and possibly reorganized in face of cortical or sensory lesions or dynamic changes of the environment. The main hypothesis of the model is that feed-forward thalamocortical connections are an adequate site of plasticity while cortico-cortical connections drive a competitive mechanism that is central in the learning process. The model relies functionally on the balance between lateral excitation and inhibition, allowing to widen or sharpen the response of the model and plays a critical role in the shaping of the receptive fields during development. This modulation of the balance may originate from at least two distinct processes at two different time scales. In the long-term, neurogenesis/neuronal death and synaptogenesis/synaptic degeneration (Edelman, 1987) are ontogenetic factors that shape cortical connectivity during development as explained in Bressler and Tognoli (2006). Synaptic density spikes during the childhood followed by a decline during adolescence and adulthood (Feinberg et al., 1990).

To further support this hypothesis, we first reproduced in this article the experimental protocol of DiCarlo et al. (1998) that has been used to characterize the structure of receptive fields (RFs) in area 3b of primary somatosensory cortex in three alert monkeys. This protocol is based on the passive stimulation of the distal finger pad using a rotating drum. This allowed the authors to show that most RFs contain a single, central region of excitation and one or more regions of inhibition. In this work, we adapted this protocol to our model and validated our results using the same modified linear regression algorithm to characterize excitatory and inhibitory components of each RF. This helped us to tune the model and we found very consistent results using a stereotyped profile for lateral connections, resulting from a fixed balance between the amount of excitation and inhibition.

We further processed our analysis by considering the dynamic modulation of the competition following a top-down signal that is supposed to originate from higher order cortical areas and has been implemented as a gain multiplication at the level of the lateral intra-cortical connections. In the short-term perspective, such modulation allows the model to give a sharper and stronger response to any stimulus. In the long term perspective, the repeated modulation of the response has a long-lasting influence onto the structure of the RFs. We hypothesized such a modulation to represent a form of somatosensory attention (spatial attention) because such modulation has been already proposed in the visual dimension as a possible mechanism for spatial attention, more specifically in area V4 (Salinas and Abbott, 1997; Salinas and Sejnowski, 2001). Indeed, attention has been mostly studied in the visual system and can be defined as a mechanism that enhances the processing of interesting (understood as behaviorally relevant) locations (spatial or featural) while darkening the rest (Posner, 1980; Treisman, 1988). The first neural correlate of that phenomenon has been discovered by Moran and Desimone (1985) in V4 where neurons respond preferentially for a given feature in their receptive fields. Since then, attentional effects have been found in each map of the ventral stream but also in the dorsal stream (area MT encoding for stimulus movement, LIP representing stimuli in a head-centered reference frame). Such attentional effects have also been identified in other modalities as well: auditory (Picton and Hillyard, 1974; Fritz et al., 2007), motor (Norman and Shallice, 1980) and somatosensory to a much lesser extent (Hsiao et al., 1993). In fact, even if the somatosensory system has been extensively studied in monkeys and rats, the nature of attentional mechanisms and how they may affect neocortical maps of somatosensory cortices remain largely unknown.

Our main hypothesis is that the modulation of a response in area 3b may be one of the core mechanism, even though the origin of the modulation signal is not detailed in this article. To test this hypothesis, we developed a specific protocol where modulation occurs only if a presented stimulus is located within a region of interest (RoI) that corresponds to the attended region and we compared results with a protocol where the region of interest is specifically trained. Results tends to highlight a prominent role of the modulation into the shrinkage of the RFs even if only the joint interaction of training and attention lead to maximal effects.

## Materials and methods

### Model

#### Finger pad

We modeled a skin patch of the index distal finger pad where Merkel's ending complex (MEC) density is known to be the highest and to convey information about touch and pressure (Pare et al., 2002). These receptors have been shown to have a sustained response to any mechanical deflection of the skin tissue. We thus considered a set of 256 receptors uniformly spread over the skin patch. When a stimulus is applied at a given position **z** of the skin patch, its mechanic property extends the pressure level to nearby locations (Goodwin et al., 1995). More formally, the response *s*_*i*_ of any receptor *i* located at **r**_**i**_ is given by the following equation:

(1) $$s_{i}(z)=\text{exp​}\left(-\frac{1}{2}\|z-r_{i}\|\right)$$

**Figure 1:**
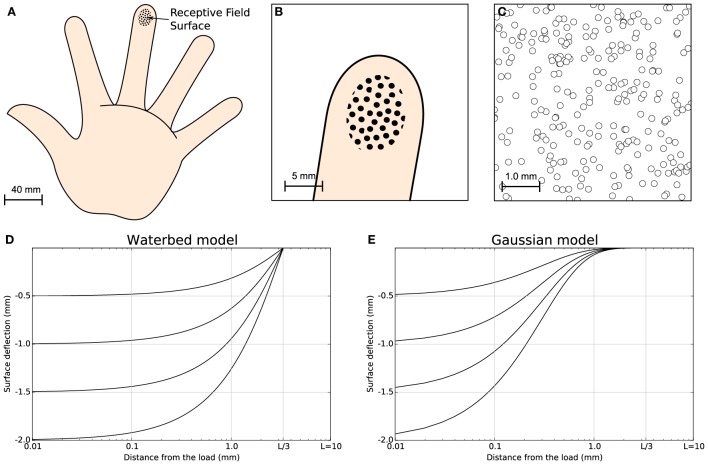
**Skin model**. The finger pad skin patch is approximately of size 25 mm^2^, using a receptor density of 10 mm^2^. It has been modeled as a planar surface and we considered 256 MEC's that are arranged in a regular grid over the whole surface with a position jitter of 5%. This results in a quasi-uniform distribution consistent with actual distribution of MEC as reported in Pare et al. (2002). **(A)** Schematic diagram of the hand. **(B)** Position and relative size of the skin patch. **(C)** Magnification of the skin patch showing MECs distribution. **(D)** Waterbed surface deflection model from Srinivasan (1989). **(E)** Gaussian surface deflection model from Detorakis and Rougier (2012). Each model predicts smaller deflection as a function of the distance from the load.

It is apparent that when a stimulus is present and its distance from the corresponding receptor tends to zero, the activity is the highest possible. On the contrary, when there is no stimulus present, the activity is zero. This model assumes a very simple correlation between the distance of the receptor to the stimulus center and its level of activity. We chose such a simple model because it eases the mathematical analysis of the model and we are not interested in the full modeling of the finger pad. More accurate models can be found in Srinivasan (1989) (waterbed model), Dandekar et al. (2003) (finite elements) and in Sripati et al. (2006b) (continuum mechanics) but we do not think using these models would fundamentally change the properties of our model (see Figure 1 for a comparison of the waterbed and Gaussian surface deflection models) since the set of 256 receptors encode a two-dimensional quantity that corresponds to the position of the stimulus.

#### Dorsal pathway

The dorsal column-medial lemniscus (DCML) pathway is the major afferent pathway for mechanosensory information and mediate tactile discrimination as well as proprioception (Purves et al., 2001). There exist several relays along this path (dorsal root ganglion, gracile and cuneate nuclei of caudal medulla and ventral posterior lateral nucleus of the thalamus) that convey information from first order neurons up to the somatosensory cortex. We modeled this complex pathway as a direct transformation of the MEC activity corresponding to the mean distance between receptors activity and the corresponding feed-forward weights. Consequently, and considering a stimulus at position **z** on the skin patch, the input *I*(**x, z**, *t*) received by a neuron **x** of SI is given by equation:

(2) $$I(x,z,t)=1-\frac{1}{n}\sum_{i=0}^{n}|s_{i}(z)-w_{f}^{i}(x,t)|$$

where *i* designates a specific skin receptor and *w*^*i*^_*f*_(**x**, *t*) is the feed-forward weight at time *t* linking receptor *i* to neuron **x**. This equation implies that any SI neuron receives input from all the skin receptors. From a neurophysiological point of view, such an assumption is valid to the extent that we considered only a small skin patch on distal finger pad. The transformation itself can be considered as the complement of the normalized distance between the set of receptors and the set of feed-forward weights. Such transformation is maximal (*I*(**x, z**) = 1) for a given stimulus **z** if ∀*i, s*_*i*_(**z**) = *w*^*i*^_*f*_(**x**, *t*). This is true because Equation (1) implies that the maximum amplitude of a stimulus is equal to one and we assumed that the feed-forward weights, *w*_*f*_, are bound between 0 and 1 and therefore the maximal value of *I*(**x, z**) = 1 and the minimum value can be *I*(**x, z**) = 0.

#### Area 3b

**Figure 2:**
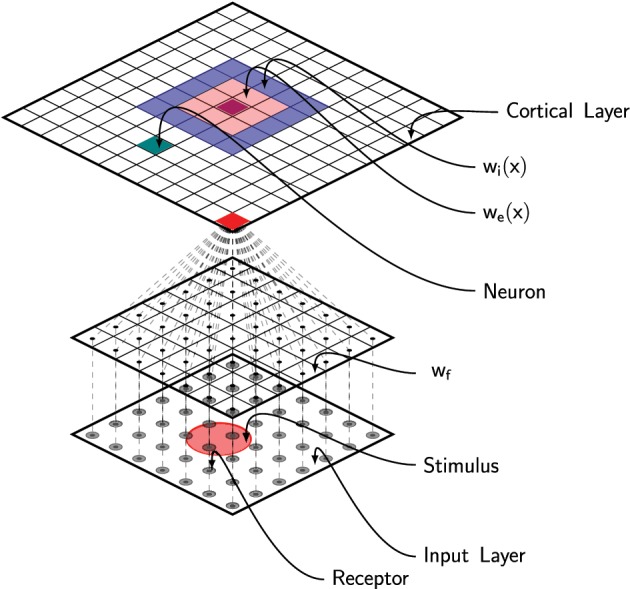
**Schematic of the full model**. Area 3b has been modeled using a neural field with lateral short-range excitation (*w*_*e*_) and long-range inhibition (*w*_*i*_). Each unit is fed with the information from all the 256 MEC receptors via feed-forward connections (*w*_*f*_).

Area 3b of the somatosensory cortex has been modeled using neural field theory (Wilson and Cowan, 1973; Amari, 1977; Taylor, 1999) which considers the cortex as a continuous surface Ω. Considering a stimulus **z**, the dynamic of the field is given by equation:

(3) $$\tau \frac{\partial u(x,t)}{\partial t}=\underset{\text{decay term}}{\underset{︸}{-u(x,t)}}\;+\underset{\text{lateral interaction}}{\underset{︸}{\int_{\Omega }^{}w_{l}(x,y)f(u(y,t))dy}}\;+\underset{\text{feed-forward input}}{\underset{︸}{I(x,z,t)}}$$

where *u*(**x**, *t*) is the membrane potential at position **x**, τ is the membrane time constant, *f* is the firing rate function, *w*_*l*_ is the lateral connections function and *I*(**x, z**, *t*) is the output from the DCLM pathway as defined in previous section (see Figure 2). The dynamic of the field is tightly linked to the lateral connections function *w*_*l*_ that defines the behavior of the field (traveling waves, spiral waves, bump solutions, see Bressloff (2011) for extensive review). In Detorakis and Rougier (2012), we defined *w*_*l*_ as a difference of Gaussian functions such as to obtain bump solutions. More precisely, we assume *w*_*l*_ is both isotropic and homogeneous (i.e., *w*_*l*_(**x, y**) = *w*_*l*_(|**x** − **y**|)) and defined as

(4) $$w_{l}(x)=w_{e}(x)-w_{i}(x)=\underset{\text{excitatory part}}{\underset{︸}{K_{e}\exp(\frac{-x^{2}}{2\sigma_{e}^{2}})}}-\underset{\text{inhibitory part}}{\underset{︸}{K_{i}\exp(\frac{-x^{2}}{2\sigma_{i}^{2}})}}$$

where (*K*_*e*_, σ_*e*_) and (*K*_*i*_, σ_*i*_) are constants that describe the extent and the strength of short-range excitation and long-range inhibition (σ_*i*_ ≫ σ_*e*_).

Learning occurs at the thalamo-cortical level using an Oja-like learning rule (proportional to a pre-synaptic measure multiplied by a post-synaptic quantity) which solves stability problems that is known to exist in the standard Hebbian learning rule see Oja, 1982). It reads:

(5) $$\frac{\partial w_{f}(x,t)}{\partial t}=\gamma \underset{\text{pre-synaptic term}}{\underset{︸}{\left(s(z)-w_{f}(x,t)\right)}}\underset{\text{post-synaptic term}}{\underset{︸}{\int_{\Omega }^{}w_{e}(|x-y|)f(u(y,t))dy}}$$

where γ is a constant learning rate. We showed in Detorakis and Rougier (2012) how this learning rule, coupled with the neural field, allow the model to self-organize and develop topological representations of the skin patch. All the details are given in Detorakis and Rougier (2012) but briefly, Equation (3) allows the model to exhibit a single bump of activity (for any input) and the learning rule (5) exploits this bump solution to promote learning at position where the excitatory part of the lateral connections function is maximal. It is to be noted that because of the pre-synaptic term and the boundedness of receptors values (i.e., are bounded between 0 and 1), feed-forward weights are also bounded between 0 and 1.

#### Gain modulation

**Figure 3:**
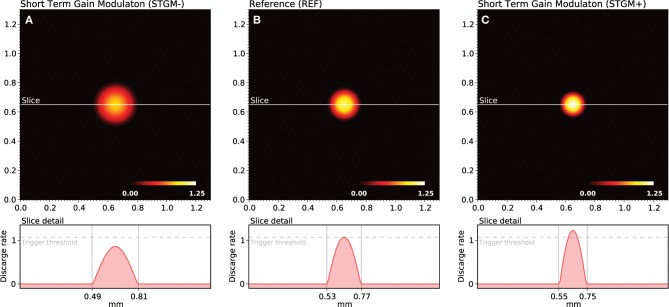
**Gain modulation**. The response of the model depends functionally on the balance between lateral excitation (gain *K*_*e*_) and inhibition (gain *K*_*i*_), allowing to widen **(A)** or sharpen **(C)** the peak of activity when a stimulus is presented. If we consider the trigger threshold to be the peak of nominal response **(B)**, the same stimulus can either trigger a sharp response or not trigger any response at all, depending on the modulation. This modulation is considered in this work as a form of somatosensory attention.

As explained earlier, the shape of the bump solution of the neural field can be controlled via lateral connections function *w*_*l*_. We have been using until now a stereotyped profile defined by the extent and the strength of short-range excitation (*K*_*e*_, σ_*e*_) and long-range inhibition (*K*_*i*_, σ_*i*_). This profile is used for the whole duration of the initial training protocol and has a direct influence on the self-organization process. We could have used instead a wider/weaker or thiner/stronger profile as shown in Figure 3 but more importantly, we can also modify it *online*, provided a signal is sent to indicate which profile is to be used for processing the next stimulus. This is what we refer as the attentional signal, originating from higher cortical areas. More precisely, we can use two parameters sets, (*K*_*e*_′, *K*_*i*_′) and (*K*_*e*_″, *K*_*i*_″), and use the first set when no attentional signal is present and the second one, when an attentional signal is present.

### Protocols

#### Initial training

Since the model initially possesses random weights, it is firstly necessary to train it in order to develop topological representations of the skin patch. We thus re-implemented the training protocol that has been used in Detorakis and Rougier (2012) and the training set is made of 50000 stimuli with random positions uniformly distributed over the whole skin patch. Each stimulus is presented once to the model and Equations (3) and (5) are evaluated simultaneously until stability is achieved, i.e., there is no noticeable difference between *u*(*t*) and *u*(*t* + δ*t*). The model is reset and another stimulus is picked up until there is no more available stimuli. A significant difference with the original model is the toric implementation of skin patch and cortical model as well. This means that any part of a stimulus that lay outside the skin patch reappears at the opposite side of the skin patch. The same holds true for the cortical sheet. This has been done to avoid any boundary effects that are known to exist in self-organization models. Once the training ends, the model has developed a topological representation of the skin patch such that two neighbor neurons on area 3b represent two neighbor location on the skin patch.

#### Drum protocol

The drum protocol is a direct adaptation of the protocol that has been used in DiCarlo et al. (1998). Authors used a cylindrical drum covered with a plastic sheet (28 × 250 mm) that possesses raised dots pattern (with a density of 10 dots per square centimeter for a total of 750 dots). The drum was mounted on a rotating drum stimulator and the orientation and the angular velocity of the drum were adjusted to produce proximal-to-distal stimulus movement at 40 mm/s across the skin surface. The drum completed 100 revolutions and the total time of simulation was 14 min and stepped a total distance of 20 mm. We adapted the drum protocol as a planar surface of size 250 × 30 mm and moved the skin patch over the full length (40 mm/s) before jumping back to the start and shifting up the patch by 200 μm. The drum surface is made of 750 uniformly distributed dots, achieving a mean density of 10 dots/cm^2^. Using a sample time step of 5 ms, the model has been fed with 120000 samples for a complete sweep of the drum surface. Activity of all neurons are recorded at once without centering the drum onto each individual receptive field.

#### RoI protocol

**Figure 4:**
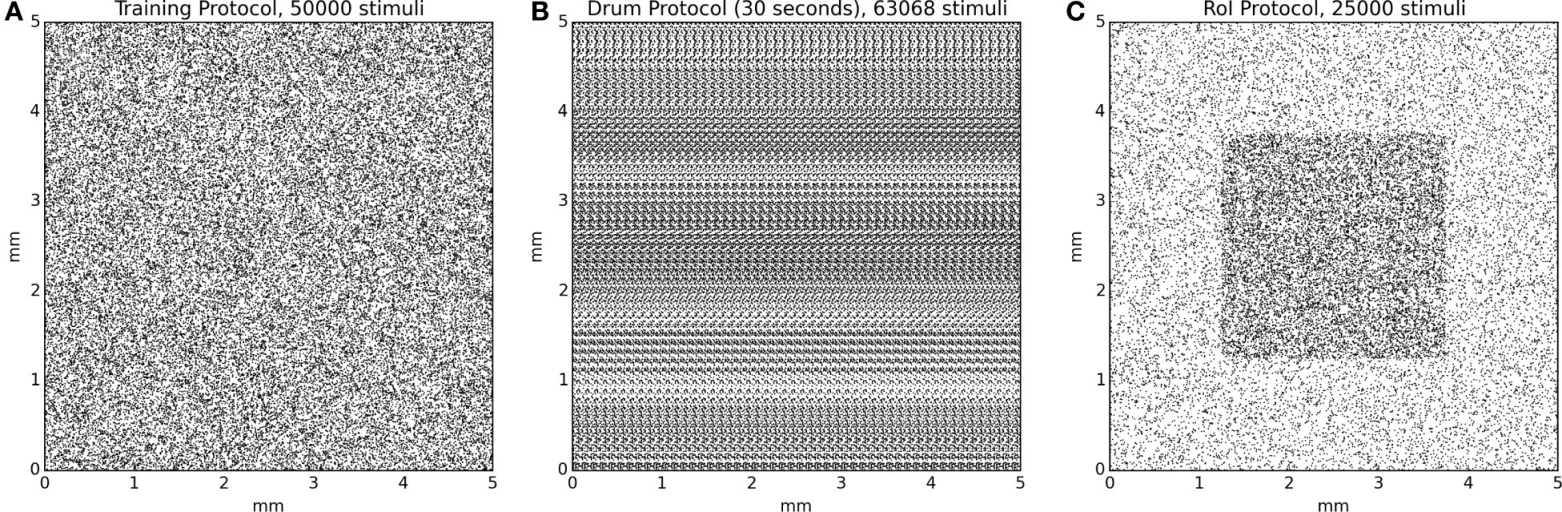
**Protocol stimuli sets**. **(A)** The training protocol set is made of 50000 stimuli distributed uniformly over the whole skin patch. At any moment, only one stimulus is presented to the model. **(B)** The drum protocol is based on a rotating drum made of 750 dots spread over the surface of the drum. The rotation of the drum makes stimuli to enter on the left side and exit on the right side of the skin patch, leading to temporal correlation between the different trials. At any moment, one to several stimuli can simultaneously stimulate the skin patch. **(C)** The RoI protocol, in the case of intensive training, is made of two sets of equal size (12500) for a total of 25000 stimuli. One set is made of stimuli exclusively located in the center of the skin patch and the other set is made of stimuli located outside this central region. This results in a higher (twofold) stimulus density in the central region. At any moment, only one stimulus is presented to the model.

We first defined an arbitrary region of interest (RoI) on the surface of skin patch whose size is one quarter of the total skin patch surface (see Figure 4C, the shaded squared area in the middle of the skin patch). For the intensive training session, we used a set of 25000 stimuli such that one out of two stimuli landed into the RoI [1 in / 1 out ratio, (see Figure 4)]. This means that the RoI, was twice more stimulated compared to the rest part of the skin patch. We presented each stimuli once to the model until no more stimuli were available. Learning occurs for the whole duration of the protocol. For the attentional experiment, we used 25000 uniformly spread stimuli over the whole skin patch. We presented each stimulus once to the model until no more stimuli were available. If a stimulus position was within the RoI, (i.e., the center of the stimulus, which is the most active zone of a stimulus) we explicitly instructed the model to attend to this stimulus by modifying the gain of the lateral connections (*K*_*e*_ and *K*_*i*_) as explained in the gain modulation section. Learning occurs for the whole duration of the protocol.

## Results

### Characterization of the RFs

**Figure 5:**
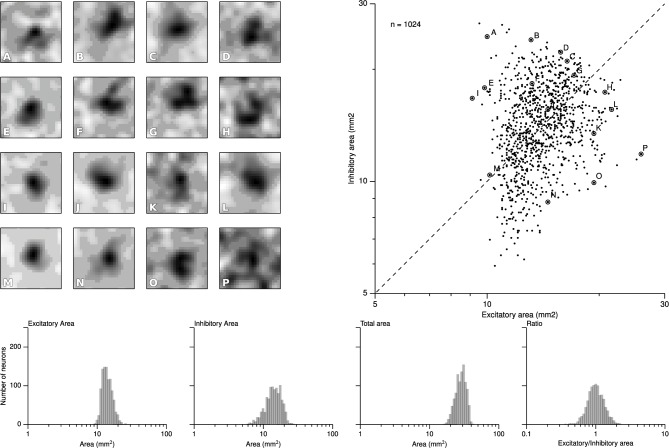
**Characterization of the ncRFs**. From the experimental drum protocol of DiCarlo et al. (1998), we recorded 120000 responses for each of the 1024 neurons of the model and we subsequently applied the same analysis with DiCarlo et al. (1998) to obtain the respective ncRF. The scatter plot on the *right* displays the balance between excitatory and inhibitory components of each ncRF. Excitatory area was measured as the total positive area in the thresholded ncRF (positive ncRF regions with values ≥10% of the peak absolute ncRF value, see Materials and Methods and Supplementary Material). Inhibitory area was measured as the total negative-thresholded ncRF area (negative ncRF regions with absolute values ≤10% of the peak absolute ncRF value). The left part of the figure **(A–P)** illustrates the diversity of ncRFs and the letter corresponds to a point in the scatter plot. The bottom row shows the distributions of the sizes of ncRFs. The y-axis indicate the number of neurons (*n* = 1024) and the x-axis, from left to right displays the excitatory area of ncRFs, the inhibitory area, the total area (is the sum of the excitatory and inhibitory areas) and the ratio of excitatory area to inhibitory one in logarithmic scale.

We first report results concerning the characterization of RF structures observed in area 3b following the exact protocol of DiCarlo et al. (1998), that was used to investigate the two-dimensional structure of area 3b neuronal non-classical receptive fields (ncRFs) in three alert monkeys (non-classical receptive fields are defined in Supplementary Material). As explained earlier, this protocol has been slightly adapted to meet the constraints of the proposed computational model architecture. Following the initial training protocol were cortical representations have been shaped (see Detorakis and Rougier, 2012), we applied the drum protocol for a total of 120000 samples (that can have multiple sites of skin patch stimulation because of the raised dot patterns). From these data, we applied the exact same linear regression algorithm proposed and used by DiCarlo et al. (1998) for the characterization of the excitatory and inhibitory components of each ncRF. More precisely, for each matrix representing an non-classical receptive field, we first convolved it with a Gaussian filter (μ = 0 and σ = 1.7) and applied a thresholding (10% of the absolute peak value) on every value. If a value was below the threshold, it was set to zero. We let each pixel of the non-classical receptive field to have at least two of the four neighbors non-zero and of the same sign such that isolated islands of positive or negative values were not allowed if they had a total area less than 0.7 mm^2^. Each time we computed a ncRF, we also computed the signal-to-noise ratio (SNR) as well as the noise index, in order to constraint them to low values (see Supplementary Material). After this preprocessing stage, we measured the respective size of excitatory (positive) and inhibitory (negative) areas. The minimum and maximum values of excitatory ncRFs were 9.12, 25.92 mm^2^, respectively for a mean size of 14.14^2^. The minimum and maximum values of inhibitory ncRFs were 5.92, 26.56 mm^2^, respectively for a mean size of 14.4^2^. Figure 5 shows the bivariate plot of excitatory vs. inhibitory area (similar results have been found by DiCarlo et al., 1998). Furthermore, a k-means classification of the ncRFs was performed on the ncRFs in order to compare the number of ncRFs classes from the model with the number of classes in DiCarlo et al. (1998). The k-means classification separated 16 different classes according to the topology of the excitatory and inhibitory areas (homogeneity = 0.39, completeness = 1.0, V-measure = 0.56). We found non-classical receptive fields whose excitatory area was surrounded by the inhibitory one as well as non-classical receptive fields whose excitatory area was facing the inhibitory area (see Figure 5). It is to be noted that Figure 5 shows a remarkable similarity with physiological results of DiCarlo et al. where most of the ncRFs are centered around a central point of 15 mm^2^ (excitatory) / 15 mm^2^ (inhibitory). The spread is larger in the case of DiCarlo but this was expected since we used a toric stimulation.

### Training the RoI

**Figure 6:**
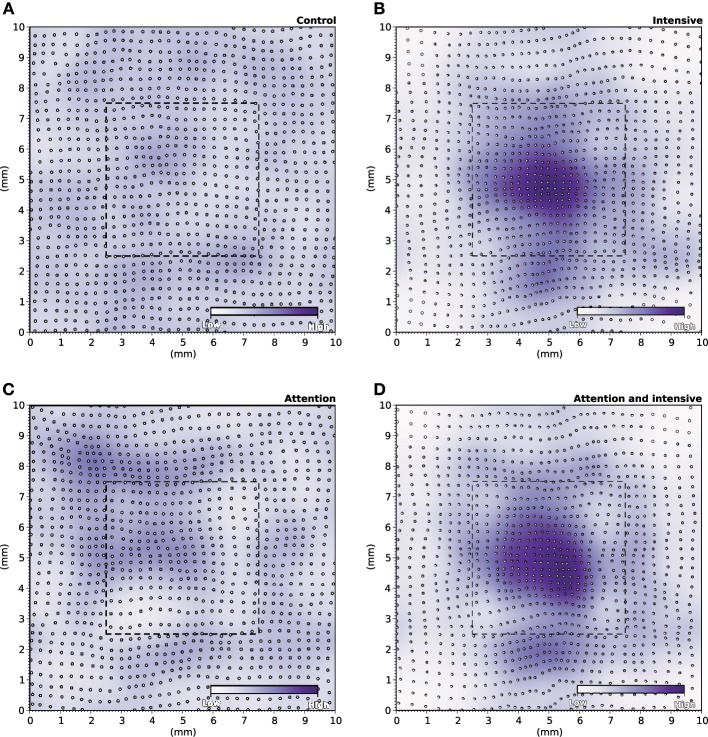
**RF Migrations**. **(A)** The distribution of RFs over the skin patch is quasi-uniformly distributed for the control. **(B)** Intensive training onto the RoI makes RFs to migrate toward the RoI leading to a higher density of RFs within the RoI. **(C)** Explicitly attending the RoI modifies only marginally the distribution of RFs that tend to remain quasi-uniformly distributed over the whole skin patch. **(D)** The joint effect of intensive training and attention leads to an even greater migration of RFs toward the RoI (compared to intensive training only).

**Figure 7:**
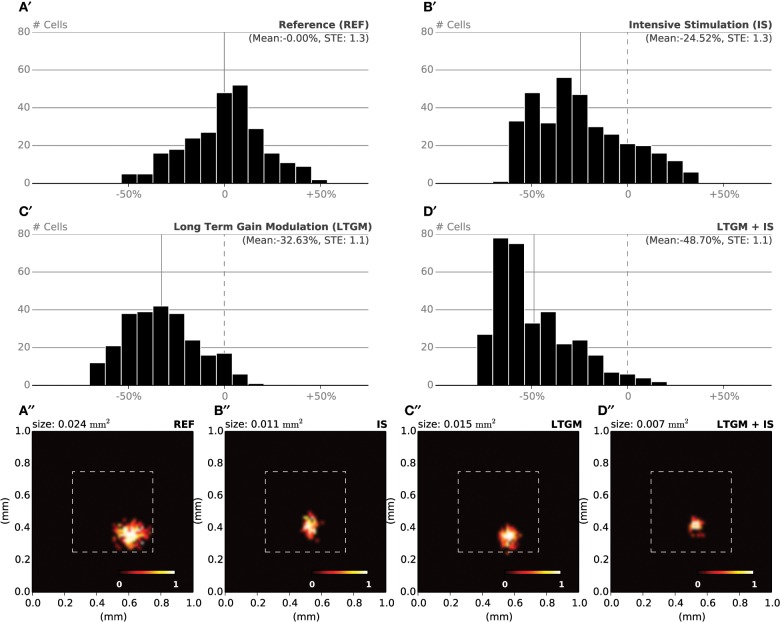
**RF Shrinkage**. **(A′)** The relative histograms of RFs sizes after initial training (50000 samples) follows a normal distribution. **(B′)** After model training, specifically in the RoI (with a 1/1 ratio) using 25000 extra samples, the mean RF size has been reduced by 25% compared to the nominal mean size. **(C′)** By sharpening the model response when a stimulus is presented within the RoI (25000 samples), the mean RF size has been reduced by 33% compared to the nominal mean size. **(D′)** The joint effect of training and modulation (25000 samples) leads to a dramatic shift in relative size of RF, with a mean size being half of the nominal mean size. **(A′′–D′′)** Receptive field of a single cell recorded at the end of each of the aforementioned experiments. The receptive field size in the attentional/intensive condition (0.007 mm^2^) has shrunk to one third of the control size (0.024 mm^2^).

During the specific training of the RoI, we considered a set of 25000 stimuli, half of them being located in the RoI. We will later refer to this as the intensive protocol. At the end of the protocol, we measured the location and the size of the classical receptive fields (cRF) or simply receptive fields (RF) (see Supplementary Material for details) and compared them to the control setup, that corresponds to the end of the nominal training period (or the start of this protocol). Figure 6B reveals a strong migration of most RF toward the RoI with an overall final density being higher in the center of the RoI in contrast to normal case illustrated in Figure 6A. We also measured RFs size at the end of the protocol and compared them with control. The control setup shows a normal distribution of sizes around a central value (2.1 mm^2^, *SD* = 0.42) while the intensive training setup leads to a significant reduction of the RFs (1.6 mm^2^, *SD* = 0.48). Overall, there has been a significant decrease in the mean size of RFs (see Figures 7B′,B″ for an isolated RF and compare with the normal case in Figures 7A′,A″, respectively). Such results are consistent with Xerri et al. (1994) that shows that intensive training over a skin area can cause the corresponding cortical territory expansion with a simultaneous shrink of receptive fields of neurons of the somatosensory cortex.

### Modulating the RoI

In order to make the model to attend to the RoI, we considered a set of 25000 stimuli, uniformly spread over the whole skin patch and we instructed the model to attend to a stimulus if this was within the RoI, i.e., using different gains for the lateral connections. The major difference compared to the intensive training experiment is the non-migration of the RFs toward the center of the RoI as shown in Figure 6C. The distribution remains actually quasi-uniform and the RoI does not benefit from significant higher density. However, the sizes of the RFs have shrunk by 33%, leading to a mean size of 1.4 mm^2^ (*SD* = 0.37). In addition, Figures 7C′,C″ show the histogram of shrinkage and the shrinkage of an individual RF, respectively. This demonstrates that migration and shrinkage of RFs are actually two distinct processes that can be (partly) separated.

### Joint effect of training and modulation

For studying the joint effect of training and modulation, we mixed the two RoI protocols and considered both a non-uniform set of 25000 stimuli, half of them being located in the RoI and we instructed the model to attend to a stimulus if it was located in the RoI. The final density of RFs shown in Figure 6D reveals a massive migration of the RFs toward the RoI with a simultaneous shrinkage in their sizes compared to the control conditions (0.71 mm^2^, *SD* = 0.04). These results point out that the combined effects of intensive training and modulation actually sum up, leading to both a massive migration and a dramatic shrinkage of RFs, down to half the nominal size (see Figures 7D′,D″).

## Discussion

Using the model presented in Detorakis and Rougier (2012), we first validated it using the protocol and neurophysiological data from DiCarlo et al. (1998). We adapted the protocol to meet computational constraints and relevant recorded data. Results clearly indicate that the model is able to capture the main aspects of the original data recorded on three alert monkeys with most of ncRFs to contain a single region of excitation and one or more regions of inhibition located on one, two, three, or all four sides of the excitatory center. This is the first, to the best of our knowledge, computational model of area 3b that is able to replicate real neurophysiological data with such accuracy even though we used a very simple model for the distal finger pad and the dorsal column-medial lemniscus, as well. This tends to confirm that the thalamo-cortical feed-forward connections are an adequate site of plasticity while cortico-cortical connections drive the competition mechanism. Furthermore, even if the present study has been circumscribed to the spatial characteristics of the receptive fields, Sripati et al. (2006a) have shown spatio-temporal receptive fields (STRF) in area 3b tend to have early excitatory region followed by in-field (replacing) greater inhibition. Authors conclude that such *greater inhibition observed in cortical STRFs points to the existence of underlying intracortical mechanisms* that is very consistent with our own hypothesis. To go further in this direction, we would need to consider finite transmission speed in cortico-cortical connections instead of instantenous connection (Hutt and Rougier, 2010).

We have also shown how this competition mechanism can be explicitly modulated by the modification of the gain at both the excitatory and inhibitory lateral connection levels. Such instructed modulation leads to receptive fields shrinkage in the region of interest while keeping intact the overall organization, with no noticeable migration of RFs. We identified such modulation as a form of spatial attention that is believed to be deployed selectively on this or that part of the body. Interestingly enough, these effects are known to occur in the visual system and a number of recent studies have identified such effects in area MT (Womelsdorf et al., 2008; Anton-Erxleben et al., 2009). More precisely, authors have shown how attention inside the cRF shrinks it, whereas directing attention next to the cRF expands it. Authors uses in their modeling work a bell shaped attentional signal while we have been using a constant attentional signal, modifying the gain for the whole population at once.

However, in the literature, the evidence for the effects of such spatial attention on SI are still contradictory. Hsiao and Vega-Bermudez (2001) has shown that attention is engaged in the modification of RFs in both primary and secondary somatosensory cortices and Braun et al. (2002) have confirmed such engagement of attention in the primary somatosensory cortex using neuroimaging techniques. However, Godde et al. (2000) claimed that attention is not critical in enhancing performance during a discrimination task even though, consecutive training or pairing stimulation (leading to co-activation) can affect the RFs. In the case of intensive training of the RoI, our model tends to suggest a large expansion of the cortical territory with a simultaneous shrinkage of the receptive fields as well as strong migration of their centers toward the RoI. These findings are still contradictory with psychophysical and neurophysiological studies such as Recanzone et al. (1992); Godde et al. (2000); Pilz et al. (2004), where authors noticed that the cortical representations undergo an expansion but at the same time RFs undergo a similar expansion. However, other neurophysiological and neuroimaging studies have shown that when cortical representations expand, RFs sizes seem to decrease (Xerri et al., 1994; Elbert et al., 1995). These latter results are also consistent with early findings of Sur et al. (1980). They found, from neurophysiological recordings and mappings that the magnification factor of cortical representations is related to the size of RFs. More precisely, the magnification factor is proportional to the size of RFs (the smaller the RFs the larger the cortical representation). Our findings tend to confirm that cortical representations in the case of intensive stimulation increase their relative size with a simultaneous RFs shrinkage. These findings indicate that there are two distinct processes at work, namely modulation and training, that are believed to be present simultaneously in most cases, while there may exist a few cases where only one process is active. This may reconcile the aforementioned contradictory results. To confirm these findings, it would thus be necessary to setup new experiments where modulation and training needs to be carefully dissociated. This can be done, for example, by precisely controlling the amount of training received by a subject and by distracting the subject such as drifting attentional process away from the primary task.

Even if our model suggests a hypothesis on how somatosensory spatial attention may modify the processing of stimulus and promote reshaping of RFs, nothing has been said so far about the exact nature, the origin and the selectivity of such attentional signal. Sarter et al. (2005) have proposed a possible circuitry involving the basal forebrain corticopetal cholinergic system since it has been observed in several studies (Donoghue and Carroll, 1987; Jimenez-Capdeville et al., 1997) that the loss of cortical cholinergic system directly impacts attentional functions. Furthermore, Juliano et al. (1991) have shown that the cholinergic depletion prevents expansion of somatosensory topographic maps, suggesting that cholinergic neurotransmitters are critical in the structure of cortical representations. Similarly, Rasmusson and Dykes (1988); Tremblay et al. (1990a,b) proposed that a cholinergic signal is responsible for the gain modulation of neuronal populations and that the co-activation of basal forebrain and the somatosensory cortex by cutaneous stimulation lead to enhanced cortical activity. Overall, such a signal may originate from a complex network involving the insular cortex, the dorsolateral prefrontal cortex, the posterior parietal cortex, the ventromedial prefrontal cortex, the posterior cingulate cortex and the anterior cingulate cortex as proposed by Menon and Uddin (2010). The main point is that the insular cortex acts as a switch between two different prefrontal networks leading to an attentional effect through saliency occurring in the anterior insular cortex. We can thus speculate that such a cholinergic signal may affect the gain of intra-cortical lateral connections and the explicit signal that has been used during the attended RoI protocol may originate from a frontal decision.

Finally, even though we hardly notice it in our everyday life, somatosensory attention plays a critical role in our perception of the outer world. For example, the contact of clothes on the skin can be largely unattended even though all body receptors are activated at once. This results from habituation and yet, it is still possible to concentrate on a specific part of the body to actually experience the contact. Such spatial selectivity is very similar to the concept of the spotlight of attention proposed by Posner (1980) in the eighties for the visual perception. At that time, authors were hypothesizing for the existence of a dedicated control mechanism even though this view was later challenged by the premotor theory of attention proposed by Rizzolatti and Craighero (1988). This later theory postulates instead that there is no need for two different mechanisms (attention and action) and has received support from several electrophysiological and brain imaging studies. However, how this theory can be adapted to somatosensory attention remains unclear. Our model cannot answer the question on the selectivity since we only used a broad and constant modulation of the model. This choice has been made because we consider a small part of SI cortex where exactly one bump of activity can exist anytime. If we were to consider a larger part of SI, where for example several digit representations would co-exist, we would need a selective attentional signal to be able to direct gain modulations to the relevant population involved in the representation of the RoI. This is quite a complex problem, since this would involve not only a sensory representation of several digits (sensory homunculus), but also a motor representation (motor homunculus) and visual information as well. This is far beyond the scope of the present work but we think this might allow for a better understanding of somatosensory attention.

### Conflict of interest statement

The authors declare that the research was conducted in the absence of any commercial or financial relationships that could be construed as a potential conflict of interest.
