# Supplementary material for: Structure of receptive fields in a computational model of area 3b of primary sensory cortex
Source: Front Comput Neurosci. 2014 Jul 28;8:76. doi: 10.3389/fncom.2014.00076 (PMC4112916; doi:10.3389/fncom.2014.00076)
Supplement: Supplementary file 1 [file Presentation1.PDF]

Structure of Receptive Fields in a Computational Model of Area 3b of Primary Sensory Cortex Supplementary Material

Georgios Is. Detorakis[1] and Nicolas P. Rougier[2,3,4,*]

[1]Laboratoire des signaux et systèmes, Supélec, Gif-sur-Yvette, France
[2]INRIA Bordeaux Sud-Ouest, Bordeaux, France
[3]LaBRI, Université de Bordeaux, Institut Polytechnique de Bordeaux, Centre National de la Recherche Scientifique, UMR 5800, Talence, France
[4]Institut des Maladies Neurodégénératives, Université de Bordeaux, Centre National de la Recherche Scientifique, UMR 5293, Bordeaux, France
[*]Corresponding author: Nicolas.Rougier@inria.fr

1 Appendix A - Detailed description of the model

1.1 Tabular description

A Model Summary

Populations	Three: receptors, thalamus and cortex
Topology	Two-dimensional, toric
Connectivity	Feed forward: one-to-all, lateral: all-to-all (including self-connections)
Neuron model	Dynamic rate model
Channel model	–
Synapse model	–
Plasticity	Oja-like learning rule
Input	Touch pressure from skin receptors
Measurements	Classical and non-classical receptive fields, topographic maps

B Topology

Name	*Type*
Receptors	Two-dimensional toric regular grid with jitter (5%)
Thalamus	None
Cortex	Two-dimensional toric regular grid

C Populations

Name	*Elements*	*Size*
Receptors	Mechanic input	256
Thalamus	Thalamic neuron	256
Cortex	Cortical neuron	32×32

D Connectivity

Name	*Source*	*Target*	*Pattern*
–	Receptors	Thalamus	Excitatory, fixed, one to one, non-plastic
W_f	Thalamus	Cortex	Excitatory, random uniform, one to all, **plastic**<br>$W_f(\mathbf{x}, t) = U(0, 1)$
W_e	Cortex	Cortex	Excitatory, Gaussian, toric, all to all, non-plastic<br>$W_e(\|\mathbf{x} - \mathbf{y})\| = K_e \exp\left(\frac{-\|\mathbf{x}-\mathbf{y}\|^2}{2\sigma_e^2}\right)$
W_i	Cortex	Cortex	Inhibitory, Gaussian, toric, all to all, non-plastic<br>$W_i(\|\mathbf{x} - \mathbf{y}\|) = K_i \exp\left(\frac{-\|\mathbf{x}-\mathbf{y}\|^2}{2\sigma_i^2}\right)$

**E1**	**Neuron Model**
Name	Thalamic
Type	Rate model
Membrane potential	$I(\mathbf{x},\mathbf{z},t) = 1 - \frac{1}{k}\sum_{i=0}^{k} \lvert s_i(\mathbf{z}) - w_f^i(\mathbf{x},t)\rvert$

**E2**	**Neuron Model**
Name	Cortical
Type	Dynamic rate model
Membrane potential	$\frac{1}{\tau}\frac{\partial U(\mathbf{x},t)}{\partial t} = -U(\mathbf{x},t) + \alpha \int_\Omega W_l(\lvert\mathbf{x}-\mathbf{y}\rvert) f(U(\mathbf{y},t))d\mathbf{y} + \alpha I(\mathbf{x},\mathbf{z},t)$

**F**	**Plasticity**
Name	*Description*
W_f	$\frac{1}{\gamma}\frac{\partial W_f(\mathbf{x},t)}{\partial t} = \gamma(s(\mathbf{z},t) - w_f(\mathbf{x},t)) \int_\Omega W_e(\lvert\mathbf{x}-\mathbf{y}\rvert) f(u(\mathbf{y},t))d\mathbf{y}$

**G**	**Input**
Type	*Description*
Mechanic	$s_i(\mathbf{z}) = \exp(-\frac{1}{2}\lVert\mathbf{z}-\mathbf{r_i}\rVert)$

**H**	**Measurements**
Classical and extended receptive fields of all cortical neurons	

Table 1: Tabular description of the model following the prescription of Nordlie et al. (2009)

1.2 Algorithms

1.2.1 Training protocol

The initial training protocol described by equations (1) to (5) can be described using the following algorithm. The full description of the learning process is given in Materials and Methods section and in Detorakis and Rougier (2012) as well.

Algorithm 1 Learning protocol

Require: $K_e, K_i, \sigma_e, \sigma_i, \gamma, \alpha, \tau, dt, S, \varepsilon$
  Compute w_e, w_i and w_l according to equation (4)
  for each stimulus $\mathbf{z}$ in S **do**
    Reset the activity $u(\mathbf{x}, 0)$
    Compute receptors activity $s(\mathbf{z})$ according to equation (1)
    Compute input activity $I(\mathbf{x}, t)$ according to equation (2)
    while $\lvert u(t) - u(t + dt)\rvert < \varepsilon$ **do**
      Update $u(\mathbf{x}, t)$ according to equation (3)
      Update $w_f(\mathbf{x}, t)$ according to equation (5)
    end while
  end for

1.2.2 RoI/modulation protocol

The RoI protocol with modulation requires a slightly different alorithm since w_e, w_i and w_l depend on the position of the stimulus.

Algorithm 2 RoI/modulation protocol

```
Require: K'_e, K'_i, K"_e, K"_i, σ_e, σ_i, γ, α, τ, dt, S, ε
  Compute w'_e, w'_i and w'_l according to equation (4)
  Compute w''_e, w''_i and w''_l according to equation (4)
  for each stimulus z in S do
    Reset the activity u(x, 0)
    Compute receptors activity s(z) according to equation (1)
    Compute input activity I(x, t) according to equation (2)
    if stimulus is inside the RoI then
      w_e, w_i, w_l ← w'_e, w'_i, w'_l
    else
      w_e, w_i, w_l ← w''_e, w''_i, w''_l
    end if
    while |u(t) − u(t + dt)| < ε do
      Update u(x, t) according to equation (3)
      Update w_f(x, t) according to equation (5)
    end while
  end for
```

1.3 Convergence

In order to measure the convergence of the learning process, we measured:

1. the evolution over time of the root mean square error (RMSE) of the feed-forward weights
2. the evolution over time of the receptive fields during the development of the topographic map and during the RoI protocol

RMSE has been measured using the following equation:

$$\mathrm{RMSE}[w_f] = \frac{1}{M}\sum_{i=0}^{M}(\hat{\mathbf{w}}_f^i - \mathbf{w}_f^i)^2, \tag{6}$$

where $\hat{\mathbf{w}}_f^i$ dsignate the final feed-forward weights and $\mathbf{w}_f^i$ the feed-forward weight at epoch i. Data is recorded every 50 epochs and RMSE is computed at the end of the simulation since it requires the final weights. Figure 1 displays RMSE for initial training and clearly shows the rapid convergence of the model on the final set of weights.

1.4 Parameters

All the parameters of the model are given in table 2 below, where n is the number of neurons of the cortex model, K_e is the excitatory gain, σ_e is the variance of the excitatory lateral connections, K_i is the inhibitory gain, σ_i is the variance of inhibitory lateral connections, α is a free scaling parameter, τ is the synapses temporal decay constant, and γ is the learning rate. For the RoI protocol, we use two sets of parameter, the first set (RoI out) is used whenever the center of a stimulus is not within the RoI while the second set (RoI in) is used each time a the center of a stimulus is within the RoI. The parameters of the model have been tuned manually and a more detailed description of the role of each parameter can be found in **Detorakis and Rougier** (2012).

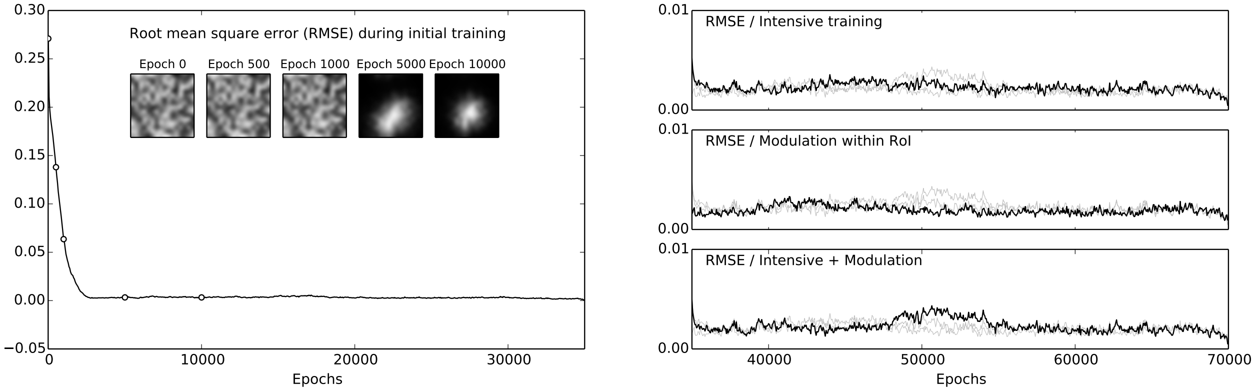

Figure 1: **Root-mean-square-error over thalamo-cortical weights.** Left. RMSE has been computed every 50 epochs during the initial training protocol and show a rapid convergence after 5000 epochs Right. RMSE has been computed for all RoI protocols to ensure no divergence occurs during training.

Protocol	n	K_e	σ_e	K_i	σ_i	α	τ	γ
Training	32×32	3.72	0.1	2.40	1.0	0.1	1.0	0.03
Drum	32×32	3.72	0.1	2.40	1.0	0.1	1.0	–
RoI out	32×32	8.02	0.1	6.10	1.0	0.1	1.0	0.03
RoI in	32×32	3.72	0.1	2.40	1.0	0.1	1.0	0.03

Table 2: Model parameters

1.5 Simulation details

Simulations were performed on a HP Z800 Workstation with 8Gb of memory. The source code of all simulations is written in Python using Numpy and Scipy scientific libraries, and it can be found at `http://webpages.lss.supelec.fr/perso/georgios.detorakis/software/index.html`. The training simulation consumes $\sim$ 45MB of physical memory and requires $\sim$ 30 minutes of real time (measured with *time* Unix command). In all simulations, we used parameters given in table 2.

2 Appendix B - Data analysis

2.1 Classical receptive fields

A classical receptive field or a receptive field is defined as the locus of the skin on which a stimulus triggers a neural response, and describes as well the impact of the stimulus to the firing rate of a neuron. A cRF is a two-dimensional surface, which slope indicates the impact of the stimulus and its location points to the skin surface which is represented by the corresponding neuron. We computed the cRF of each neuron using a grid of $p \times p$ regularly spaced stimuli over the normalized skin patch. For each stimulus and after the model has converged, the activity of each neuron of the model has been recorded and aggregated into a $p \times p$ matrix that was identified as the cRF of the neuron. cRFs have been further charaterized by determining their center of mass and size. The center of mass $\mathcal{C}$ is computed according to the following equation:

$$\mathcal{C} = \frac{\sum_{i=0}^{p^2} \mathcal{V}_i \mathbf{s_i}}{\sum_{i=0}^{p^2} \mathcal{V}_i} \tag{7}$$

where $\mathbf{s}_i$ is the position of a stimulus (i in $[1, p^2]$) and $\mathcal{V}_i$ is the related activity of the neuron (activity of the neuron when stimulus $\mathbf{s}_i$ is presented). The cRF area is computed using a normalized sum of the elements of the cRF that are grater than a threshold value (< 0.05). Using these two informations, we can build plots of cRFs lying at their respective position on the skin patch with high precision. We can therefore qualify the topographic organization of the cRF and, when relevant, we can define the migration as well as size modification. The density of cRF over the skin patch was evaluated using a Gaussian filter with standard deviation of 0.5 in each dimension.

2.2 Non-classical Receptive fields

We define non-classical receptive fields as the weights $\mathbf{b}$ that minimize the error between the predicted firing rates of neurons and the observed ones. Such non-classical receptive fields reveal what is the optimal stimulus and how stimulus affect the firing rates of neurons. We computed non-classical receptive fields (ncRFs as opposed to cRFs) using the protocol and method (analogue to reverse correlation) defined in DiCarlo et al. (1998). Therefore, the predicted impulse rate r_p of a neuron in response to the nth stimulus is defined to be the sum of the effects of each skin subregion to the neuron (see drum protocol for more details). Hence,

$$\mathbf{r}_p = b_0 + \sum_{i=1}^{p} b_i x_i(n) \tag{8}$$

where n is the number of the stimuli, p is the number of the different skin subregions, x_i is the stimulus relief, b_0 is the background firing rate and b_i is the strength of the effect of a dot (since the stimulus each time is a random dot pattern). Rewriting equation (8) in vector form we have,

$$\mathbf{r}_p = \mathbf{Xb}$$

where $\mathbf{r}$ is a $n \times 1$ vector of the firing rates, $\mathbf{X}$ is a $n \times p$ matrix with values of ones in the first column and the stimuli in the remaining columns. Finally $\mathbf{b}$ is a $p \times 1$ vector, which contains the weights of the effect of a stimulus to the neuron. This term is actually the non-classical receptive field of a neuron, since its values indicate the way that a stimulus affect the firing rate properties of a neuron. In addition, $\mathbf{b}$ can be used in order to investigate further the optimality of each stimulus, in terms of neural responses. Besides the predicted firing rates, we had also a vector of observed discharge rates for every neuron, $\mathbf{r}_o$.

In order to compute the vector $\mathbf{b}$, which minimizes the mean-squared error between $\mathbf{r}_p$ and $\mathbf{r}_o$, we solved the linear normal equations $\mathbf{X}^T\mathbf{X}\mathbf{b} = \mathbf{X}^T\mathbf{r}_o$ by inverting the stimulus autocorrelation matrix,

$$\mathbf{b} = (\mathbf{X}^T\mathbf{X})^{-1}\mathbf{X}^T\mathbf{r}_o \tag{9}$$

This method allows to compute the non-classical receptive fields, $\mathbf{b}$ for all the neurons at one and indicates skin area that contribute positively to the firing rate (excitatory part) or negatively (inhibitory part). Throughout of this work anytime we refer to the non-classical receptive fields we mean the optimal vectors $\mathbf{b}$. For more details about this method refer to **DiCarlo et al.** (1998).

Signal-to-noise ratio (SNR) and Noise Index (NI)

When we applied the threshold method described in the main text, we also measured the SNR and the Noise Index. From signal processing definition, noise is defined to be the residual of the subtraction between an original signal s and the filtered one $\bar{s}$ (see main text for more details about filtering and thresholding method). In our case, the original signal is the receptive field and the filtered signal is the receptive field after the application of a Gaussian filter with zero mean and variance $1,7$. SNR is given by SNR$=10\log_{10}\left(\frac{S}{N}\right)$, where S is the power of the original signal s and N is the noise power. We measured the SNR for each RF and we found that the mean SNR was 8 (S.D.=1.5).

In addition, and in accordance with **DiCarlo et al.** (1998), we computed the Noise Index (NI) according to equation $NI = 100\frac{\text{Var}[noise]}{\max\{|s|\}}$. NI is given in terms of the absolute peak of the original signal s. We measured the Noise Index for each of the RF, finding a mean value of 1.5% (S.D.=0.01). Conclusively, both measures SNR and Noise Index pinpoint that the noise was eliminated.

2.3 Relative histogram of cRFS

Histograms of receptive field sizes have been made relatively to the control case in order to underline the various changes. Considering the mean size M (over the 32×32 RFs) of the control and a set $\{X_i\}_n$ of observations, histogram were built using a set $\{Y_i\}_n$ such that $Y_i = 100(X_i - m)/m$ with a fixed size bins of width 8%. Any bin with negative abscissa indicated a shrinking in RF size while positive abscissa indicated an expansion.

2.4 Cortical representation

Cortical representation is defined as the area of the cortical surface that respond when a stimulus is present within the RoI. Using the RoI protocol, we recorded the two-dimensional activation of the whole cortical model if, and only if, the stimulus center was located within the RoI (roughly half of the 25,000 stimuli). We summed these activations and normalized the result to get values in the [0,1] interval.

2.5 Spatial event plot

During the execution of the drum protocol, the alignment of the stimulus and the responses of a specific neuron was done by applying the spatial event plot (SEP) method described in **Phillips et al.** (1988) and used by **DiCarlo et al.** (1998). The latter used a variation of SEP method in order to achieve better resolution. Since we deal with a computational model, the first version of the method was used because it is easier to achieve the alignment of the stimulus and the neural response. We isolated the receptive field of a specific neuron and applied the drum protocol (see main text for more details), recording all the responses of that neuron. The stimulus and the responses of the neuron were recorded for later processing. We processed the array of the responses by assigning a one to the element of array which contained an evoked response and a zero otherwise. Because the events array is a one-dimensional, we had to transform it into a two-dimensional one in order to fit the drum (input space). Therefore, we started from the lower right

corner (where the first stimulus was applied according to the drum protocol) and we continuously skimmed the array. Every time the drum swept was completed, we moved the next line of the array above the current one. At the end of this process, we obtained a spatial event plot, as it is depicted in figure 2B. In addition to the SEP, we also convolved the stimuli (dot patterns) with the non-classical receptive field of a neuron such as to obtain a prediction of the responses of the neuron to each stimulus. This method leads to the results shown in figure 2A for a specific neuron.

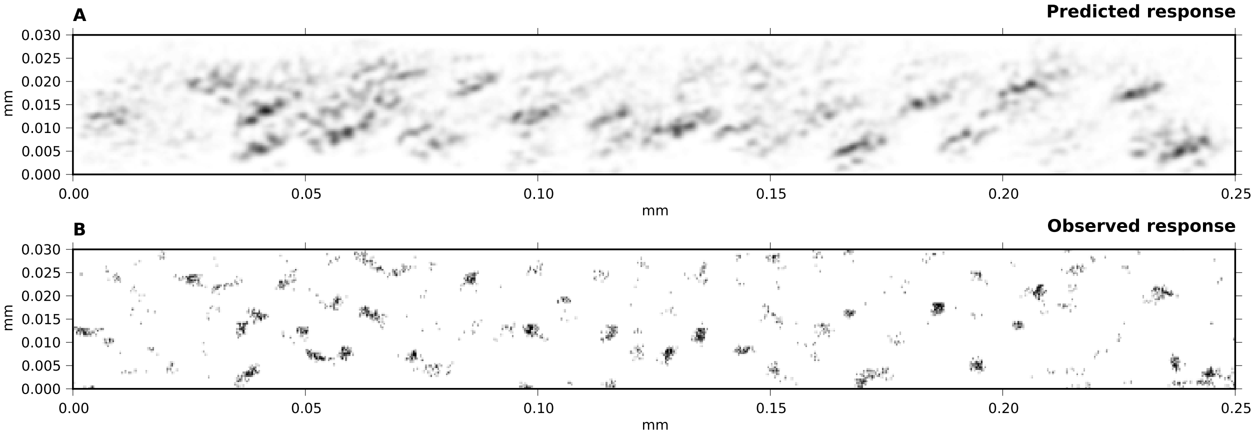

Figure 2: **Observed and predicted responses.** A. Neural impulse rates of the neuron $(20, 16)$. In order to obtain this plot, we convolved the ncRF of the neuron with the random dot stimulus pattern. B. Spatial event plot of neuron $(20, 16)$. Each dot represents the position where a stimulus triggers a response for the neuron. The axis x and y represent the length and the width of the drum (drum protocol, see the main text) in mm, respectively.

References

Detorakis, G. and Rougier, N. (2012), A neural field model of the somatosensory cortex: Formation, maintenance and reorganization of ordered topographic maps, *PloS one*, 7, 7, e40257

DiCarlo, J. J., Johnson, K. O., and Hsiao, S. S. (1998), Structure of receptive fields in area 3b of primary somatosensory cortex in the alert monkey., *Journal of neuroscience*, 18, 7, 2626–2645

Nordlie, E., Gewaltig, M.-O., and Plesser, H. E. (2009), Towards reproducible descriptions of neuronal network models, *PLoS Comput Biol*, 5, 8, e1000456, doi:10.1371/journal.pcbi.1000456

Phillips, J., Johnson, K., and Hsiao, S. (1988), Spatial pattern representation and transformation in monkey somatosensory cortex, *Proceedings of the National Academy of Sciences*, 85, 4, 1317–1321
